# Describing endosymbiont–host interactions within the parasitism–mutualism continuum

**DOI:** 10.1002/ece3.11705

**Published:** 2024-07-04

**Authors:** Ary A. Hoffmann, Brandon S. Cooper

**Affiliations:** ^1^ Pest and Environmental Adaptation Research Group, School of BioSciences, Bio21 Institute University of Melbourne Parkville Victoria Australia; ^2^ Division of Biological Sciences University of Montana Missoula Montana USA

**Keywords:** addiction, cytoplasmic incompatibility, endosymbiosis, host–microbe interactions, infection, manipulation

## Abstract

Endosymbionts are widespread in arthropods, living in host cells with effects that extend from parasitic to mutualistic. Newly acquired endosymbionts tend to be parasitic, but vertical transmission favors coevolution toward mutualism, with hosts sometimes developing dependency. Endosymbionts negatively affecting host fitness may still spread by impacting host reproductive traits, referred to as reproductive “manipulation,” although costs for hosts are often assumed rather than demonstrated. For cytoplasmic incompatibility (CI) that involves endosymbiont‐mediated embryo death, theory predicts directional shifts away from “manipulation” toward reduced CI strength; moreover, CI‐causing endosymbionts need to increase host fitness to initially spread. In nature, endosymbiont–host interactions and dynamics are complex, often depending on environmental conditions and evolutionary history. We advocate for capturing this complexity through appropriate datasets, rather than relying on terms like “manipulation.” Such imprecision can lead to the misclassification of endosymbionts along the parasitism–mutualism continuum.

## Graphical abstract

Bacteria that live inside the cells of arthropod hosts (“endosymbionts”) are gaining increasing interest as targets for pest control and for reducing the ability of arthropod vectors to transmit animal and plant viruses that cause diseases. While these endosymbionts can spread into host populations by impacting the reproduction of their hosts in different ways (typically referred to as “manipulating” their hosts), such anthropomorphic terms detract from the diverse and complex interactions of endosymbionts with their hosts which can make them parasitic in some circumstances and beneficial in others, in turn affecting their usefulness in applied contexts. We therefore advocate describing endosymbiont–host interactions by using terms that link directly to traits, and we suggest avoiding terms like “manipulation”, “hijacking,” and “addiction” as much as possible.

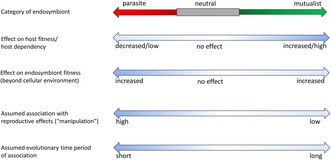

## 1 INTRODUCTION

Bacterial endosymbionts are diverse and include genera composed of only host‐dependent endosymbionts (such as *Wolbachia*, *Buchnera*, and *Cardinium*) to others that also include members that may live independently of host cells such as *Arsenophonus*, *Serratia*, and *Sodalis* (Chari et al., 2015; Gherna et al., 1991; Williams et al., 2022). Once established in host lineages, endosymbionts are typically considered as vertically transmitted through the female's germline (Bright & Bulgheresi, 2010; Koga et al., 2012; Radousky et al., 2023), although non‐ovarial forms include transmission through plants (Gu et al., 2023; Lemaire et al., 2012) and predators/parasitoids like some wasps (Heyworth & Ferrari, 2016). Vertical transmission is critical for the trajectory of endosymbiont–host associations since it can directly favor evolution toward mutualism. Endosymbionts are usually separated from other microbial symbionts and gut bacteria, which may have both vertical and horizontal transmission modes within host species, with vertical routes that can be outside of ovarial‐based transmission (Bright & Bulgheresi, 2010). Even in the case of strict vertical transmission within host species, transmission rates may vary across environmental or genetic contexts, leading to changes in endosymbiont frequencies in host populations (Carrington et al., 2011; Hague et al., 2022).

**FIGURE 1:**
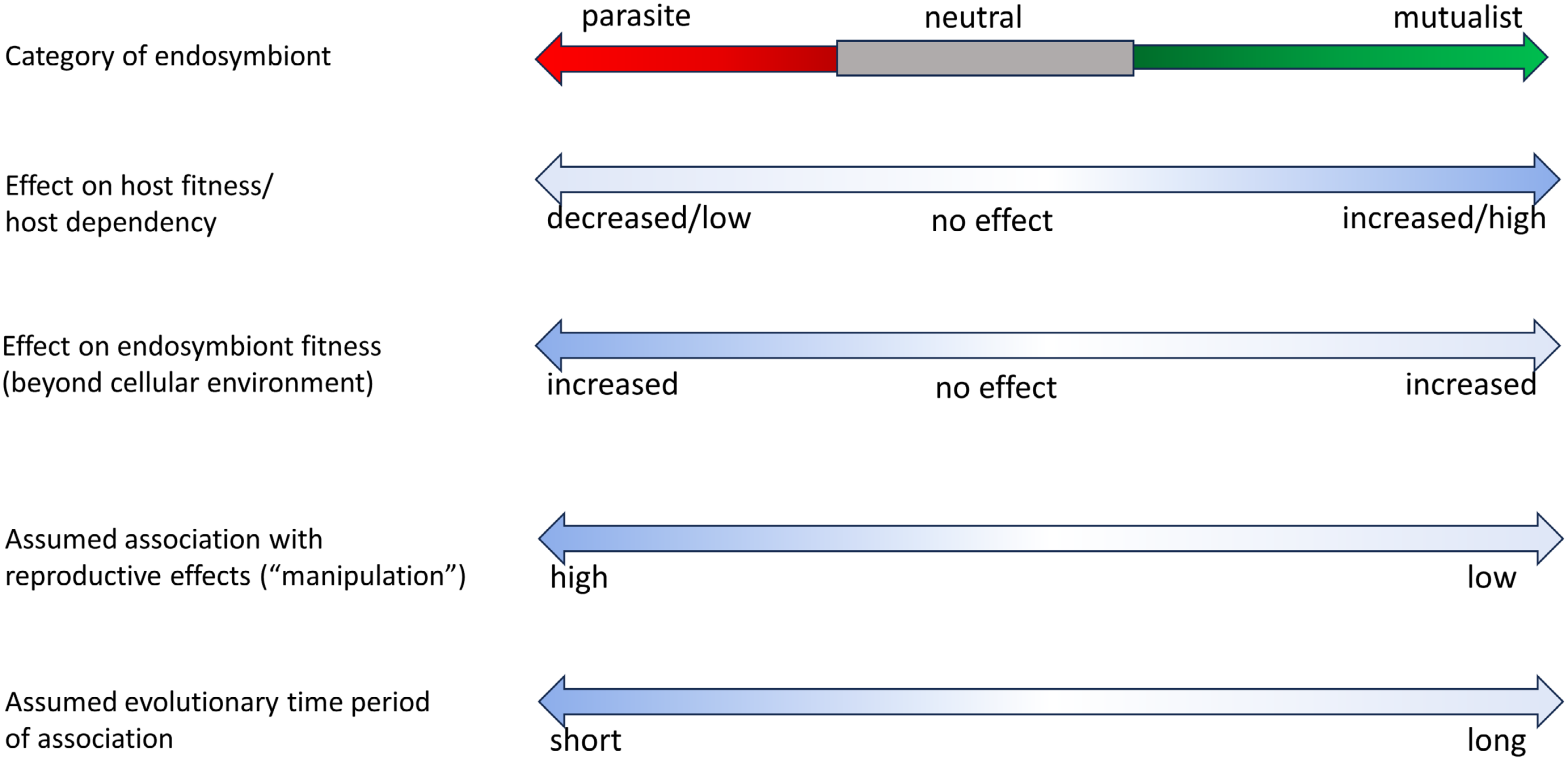
Categorization of endosymbionts and traits affected by the endosymbionts from the perspective of reproductive effects (often described as “manipulations” in the literature) and fitness effects on hosts. Mutualism implies that both parties benefit but endosymbionts also benefit by being parasitic. Reproductive trait effects are discussed in the text. Theory predicts that new deleterious associations may evolve toward mutualism in the longer term.

**FIGURE 2:**
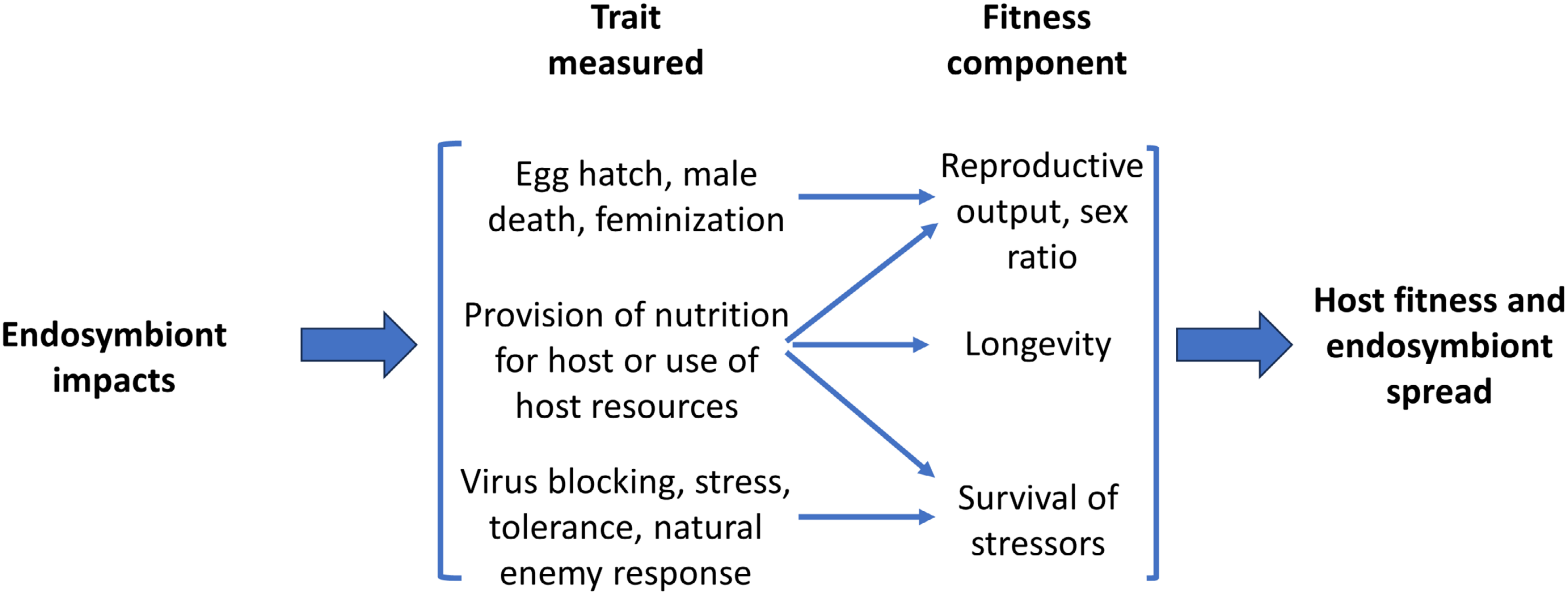
Interaction between endosymbionts and hosts assessed through (host) traits affected and their influence on (host) fitness components which impacts total host fitness and endosymbiont spread. Traits affected by endosymbionts may have positive or negative effects on host fitness components (which in turn are influenced by environmental conditions including biotic and abiotic stressors). The sum of these effects dictates where endosymbionts fall along the parasite‐mutualist continuum.

Endosymbiont effects on host traits dictate where endosymbionts fall along the parasite to mutualist continuum, influencing the fitness of endosymbionts (and their frequency in host populations) as well as host fitness (Figures 1 and 2). We use the terms obligate and facultative here to describe associations where hosts do or do not require the endosymbiont to survive, respectively. Newly acquired facultative endosymbionts are often seen as parasites, gaining resources from their hosts to increase their own fitness (Figures 1 and 2). These include most deliberate artificial transinfections of endosymbionts into new hosts, such as *Wolbachia* in *Aedes* mosquitoes for biocontrol of arboviruses (Nazni et al., 2019; Ross et al., 2019; Utarini et al., 2021), *Wolbachia* in planthoppers for disruption of rice‐virus transmission (Gong et al., 2020), and *Rickettsiella* in aphids for decreasing host fitness (Gu et al., 2023). Specific fitness effects of natural facultative associations vary but may include contributing to host nutrition, and in some cases reducing the impact of external threats to the host such as those associated with pesticides (Kikuchi et al., 2012), heat stress (Heyworth & Ferrari, 2016), and predators/parasitoids (Shigenobu & Wilson, 2011; see Table 1). For example, *Spiroplasma* bacteria reduce the impacts of nematode, insect, and fungal organisms on hosts (Ballinger & Perlman, 2019). In this case, the benefits to the host are obvious but the endosymbiont benefits are restricted to enhancing the likely persistence of the host, and thus, their own transmission.

**TABLE 1:** Examples of trait effects of endosymbionts and whether these are typically regarded as contributing to the endosymbiont being more toward the parasitic or mutualistic end of the continuum in Figure 1.

Traits	Bacteria that have been linked to these traits^a^	Impact on position in parasitism–mutualism continuum	References
Cytoplasmic incompatibility (CI)	*Wolbachia*, *Cardinium*, *Rickettsiella*, *Spiroplasma*, and *Mesenet*	Parasite	Hunter et al. (2003), Rosenwald et al. (2020) and Takano et al. (2021)
Male killing (MK)	*Spiroplasma*, *Wolbachia*, *Rickettsia*	Parasite to mutualist	Jiggins et al. (2000) and Richardson et al. (2023)
Parthenogenesis	*Rickettsia*, *Wolbachia*	Neutral	Aguin‐Pombo et al. (2021) and Ebrahimi et al. (2019)
Nutrition provision directly or through plant interactions	*Buchnera*, *Serratia*, *Coxiella*, *Wolbachia*, and many others with complex interactions possible	Mutualist	Ben‐Yosef et al. (2020) and Manzano‐Marın et al. (2020)
Life history cost (not male killing)	*Rickettsiella*, *Wolbachia*, and many others	Parasite	Gu et al. (2023) and Zhu et al. (2021)
Life history benefit (with unclear basis)	*Cardinium*, *Wolbachia*, and others	Mutualist	Kanyile et al. (2023) and Katlav et al. (2022)
Increased survival from natural enemy attack	*Hamiltonella*, *Rickettsia*, *Regiella*, and others	Mutualist	Fan et al. (2022) and Jamin and Vorburger (2019)
Reduced virus transmission	*Wolbachia*, *Regiella*, and others	Neutral	Bruner‐Montero and Jiggins (2023) and Higashi et al. (2023)
Increased virus transmission	*Rickettsia*, *Buchnera*, *Arsenophonus*, and others	Neutral	Kaur, Singh and Joshi (2022) and Shi et al. (2021)

^a^ Some bacteria appear in multiple places (e.g., *Wolbachia*, *Regiella*, *Rickettsia*) because (1) different strains of the bacteria in the same host can lead to different traits being affected, (2) the host can influence the traits expressed, and (3) the same bacterial strain can influence multiple traits.

Traits affected by facultative endosymbionts often lead to costs for the host that may push the endosymbiont toward the parasitism end of the continuum in Figure 1. Benefits to hosts are often condition‐ and environment‐dependent: There is little point in having an endosymbiont‐based defense system if natural enemies are absent, for example. Similarly, endosymbiont costs can be context‐dependent, such as the negative impacts of some endosymbionts on survival of diapausing or quiescing life stages that are only expressed under extreme conditions (Kriesner et al., 2016). These costs are not static and can evolve and presumably diminish as the time of the endosymbiosis extends. Interactions may even evolve to switch from costly to beneficial, as observed for *w*Ri *Wolbachia* that rapidly evolved from decreasing to increasing *Drosophila simulans* fecundity in a few decades (Weeks et al., 2007).

High endosymbiont densities in host tissues are often seen as having particularly strong negative effects that can even kill hosts (Min & Benzer, 1997), although high densities in reproductive tissues can be important for transmission and there can be a conflict between hosts and endosymbionts in terms of optimal density effects (Parker, 2021; Radousky et al., 2023). The fitness effects of endosymbiont densities may also be context, and in this case, host‐tissue dependent (Neiers et al., 2021). Over‐proliferation in some tissues may harm a host (Carrington et al., 2009), while high densities in other tissues or contexts that are well regulated by the host may be crucial for obtaining other (positive) endosymbiont effects (Whittle et al., 2021). Only a few genomic changes may be required to influence endosymbiont density and tissue distribution (Chrostek & Teixeira, 2015), providing the opportunity for rapid shifts in endosymbiont effects on host traits, host and endosymbiont fitness, and endosymbiont transmission.

Host traits may also become dependent on, but not improved or expanded by, endosymbionts they carry. This has been described in the literature as host “addiction” (Hammer, 2023; Moran, 2002). In contrast, endosymbionts with longer host associations (often obligate) may coevolve with their hosts toward mutualism. As with facultative associations, the fitness effects of obligate associations vary (Table 1), but several examples of endosymbionts synthesizing vitamins, essential amino acids, and other compounds are well understood in multiple systems (McCutcheon & Moran, 2012). The classic endosymbiont in this respect is *Buchnera* that has codiverged with aphid hosts for ~200 MY (Moran et al., 1993) and has become essential to aphids in partitioning amino acids that enable their hosts to leverage low‐nutrient phloem sap (Baumann, 2005; Douglas, 1998; Hansen & Moran, 2011). These endosymbionts tend to be (accurately) described as beneficial to hosts.

Characterizations of facultative endosymbionts as parasites or mutualists (or even neutral for that matter) based on a few endosymbiont trait effects are unlikely to accurately define them (Figure 2). This is similar to the problem of measuring fitness—partial measures (i.e., fitness components) may be negatively correlated and fail to serve as accurate proxies for total fitness (Flatt, 2020; Fowler et al., 1997). The literature is full of examples of endosymbiosis researchers (including us) using terms like “manipulation” that imply directionality in ways that can unintentionally misclassify endosymbiont associations within the parasitism–mutualism continuum in the absence of necessary data. We discuss this issue and argue for neutral terminology when possible that focuses on accurately describing the relevant effects and phenotypes that have been characterized in a more objective way. Precisely describing observations in the context of the host and its environment is central to our argument.

## 2 ORIGIN AND TRANSFER OF ENDOSYMBIONTS AND IMPLICATIONS FOR THEIR PLACEMENT WITHIN THE PARASITISM–MUTUALISM CONTINUUM

The precise origins of any specific endosymbiont are difficult to determine, but all endosymbionts ultimately derive from free‐living ancestors. Many interactions between potential eukaryotic hosts and microbes are ephemeral, taking place in a single generation, with microbes invading hosts but failing to transmit to the next generation. Those interactions that persist are historically considered symbioses (De Bary, 1879), regardless of their fitness effects (McCutcheon, 2021), although contemporary dictionary definitions emphasize fitness benefits to both parties. The direct ancestors of contemporary endosymbionts are likely of parasitic origin, stemming from horizontally transmitted insect pathogens that evolved to infect host cells at certain life stages (Ewald, 1987; Sachs, Skophammer & Regus, 2011). Pathogen virulence varies (Casadevall & Pirofski, 2001)—including some infections that are relatively mild and persistent—and, as well documented in human pathogens; virulence also depends critically on the response of the host (Casadevall & Pirofski, 1999). By evolving some degree of vertical transmission, these persistent bacteria can transition to stable endosymbiosis (McCutcheon et al., 2019; Sachs, Skophammer & Regus, 2011; Suh et al., 2001). Through time, facultative endosymbionts that persist may evolve to benefit the fitness of their hosts, enhance their own vertical transmission, and make the transition to obligate associations where they move further toward the mutualism end of the continuum.

Obligate endosymbionts are typically restricted to a specialized organ (e.g., a bacteriome) and may rapidly lose genes associated with living outside of host cells and bodies (McCutcheon & Moran, 2012; Moran et al., 2008), although the specific genes that are lost is context‐dependent (Degnan et al., 2009). Gene loss and associated host‐dependence makes the transition to obligate association dramatic and permanent for the endosymbiont (Husnik & Keeling, 2019), although not all obligate endosymbionts undergo a process of gene loss (Kampfraath et al., 2019). Nevertheless, obligate relationships may be complex and dynamic, as in mealybugs that may have either a single *Tremblaya* endosymbiont, or multiple endosymbionts that contribute to host nutrition (Garber et al., 2021). Systems with obligate endosymbionts may also have other facultative endosymbionts (Moran et al., 2008), and very divergent endosymbionts may contribute in similar ways to host functions. For example, a fungal endosymbiont that is closely related to pathogenic free‐living *Ophiocordyceps* seems to have replaced a bacterial endosymbiont in some cicadas (Matsuura et al., 2018).

Established endosymbiont associations in one host species may transfer to others. Host lineages may simply acquire an endosymbiont from common ancestors during host speciation. These cases not only tend to involve longer term and coevolved obligate associations but may also involve facultative endosymbionts (Moran et al., 2008; Raychoudhury et al., 2009). Unlike obligate associations that tend to evolve metabolic specificity to specific hosts (McCutcheon, 2021), newly formed facultative associations may continue to transfer horizontally among host species, but the capacity to switch hosts need not be restricted to evolutionarily young endosymbionts. For example, *Wolbachia* associations with insects and other arthropods are tens to hundreds of millions of years old, but *Wolbachia* regularly transfer horizontally between diverse hosts (Raychoudhury et al., 2009; Shropshire et al., 2023; Turelli et al., 2018). This host switching occurs via mostly unknown ecological mechanisms (Ahmed et al., 2016; Zhao et al., 2021), although plants and parasitoids are plausible vectors (Ahmed et al., 2015; Chrostek et al., 2017; Vavre et al., 1999). Clearly *Wolbachia* interactions with conserved cellular processes ultimately underlie their successful association with diverse hosts and status as the most common endosymbionts in nature (Serbus & Sullivan, 2007; Weinert et al., 2015).

How and when hosts acquire their endosymbionts influences how we describe them and where they fall along the parasitism–mutualism continuum (Ewald, 1987). While very newly acquired endosymbionts tend to resemble pathogens infecting hosts, relatively old and often obligate associations are predicted to have coevolved toward mutualism (Figure 1). The case of *Wolbachia* is again particularly interesting, since it serves as an exception. *Wolbachia* associations with insects are relatively old, yet examples of cladogenic transfer during host speciation are relatively rare in the literature (Gerth & Bleidorn, 2016; Raychoudhury et al., 2009), while examples of rapid host switching are common (Cooper et al., 2019; Sanaei et al., 2021; Shropshire et al., 2023; Turelli et al., 2018). Despite this, *Wolbachia* are predicted to increase host fitness to spread within host populations (Hoffmann et al., 1990). Thus, a necessary conclusion is that while most contemporary *Wolbachia*‐host associations are evolutionarily short‐lived in any host lineage, many *Wolbachia* still increase host fitness, at least in *Drosophila*, butterflies, and other groups where horizontal transfer is common.

Understanding and accurately describing the specific host traits affected by endosymbionts (in particular, facultative endosymbionts) and their contributions to field fitness is a crucial future area of basic and applied research (Figure 2). The range of potential effects is wide (Table 1), but for most endosymbiont–host systems, the relative contributions of fitness components measured in laboratory experiments to field fitness are often unresolved (e.g. Smith et al., 2021; Zepeda‐Paulo et al., 2017). Thus, while theory can clearly establish predictions (e.g., vertical transmission leads to coevolution toward mutualism), unique aspects of particular systems (e.g., mixed among‐species transfer modes) complicate placement within the parasitism–mutualism continuum and raise questions about the contributions of particular endosymbiont effects on hosts to their overall influence on host fitness.

While endosymbiont effects on hosts are diverse, here we focus on endosymbiont effects described as “manipulating” host reproduction to spread as an example. The classic reproductive “manipulation” is endosymbiont‐induced cytoplasmic incompatibility (CI) that reduces the egg hatch of embryos fertilized by males carrying the endosymbiont (Yen & Barr, 1971). Endosymbionts expressing CI and other “manipulation” traits are regularly considered “manipulators,” regardless of whether the endosymbiont has positive or negative effects on host fitness. We discuss this below to illustrate the difficulty and importance of accurately describing endosymbiont–host interactions.

## 3 “MANIPULATING” HOSTS

Where negative fitness effects are strong and persistent, facultative endosymbionts should be lost from populations. Indeed, most interactions are likely occurring on the order of single generations, whereby an endosymbiont is acquired and lost due to negative effects on hosts or random drift. However, many endosymbionts affect reproductive traits of their hosts which are typically interpreted in terms of reproductive “manipulation” of the host to enhance the persistence and spread of the endosymbiont at the cost of the host. The term “manipulation” may have initially been a useful way of communicating the interesting effects that endosymbionts like *Wolbachia* and *Cardinium* have on host reproduction to broad audiences including the public and students (Werren et al., 2008). However, widespread usage of “manipulation” in the case of traits and “manipulator” in the case of endosymbionts expressing these traits may now detract from the complexity of factors and contexts influencing endosymbiont dynamics.

Currently in the literature nearly all cases of these endosymbiont‐expressed reproductive effects are assumed to represent examples of them “manipulating” (or “hijacking”) hosts (e.g., Dijksterhuis, 2023; Fukui et al., 2015; Katsuma et al., 2022) but often without specific data to show that the reproductive effects are detrimental to the host while enhancing endosymbiont fitness (Figure 2). Relevant effects include not only CI but also the differential death of male embryos and young offspring (male killing) (Hurst et al., 1999), the induction of parthenogenesis (Stouthamer et al., 1993), and the conversion of male offspring to females (Kageyama et al., 2017).

CI is the most well‐studied reproductive “manipulations.” CI caused by divergent endosymbionts has been observed in at least 10 arthropod host orders (Shropshire et al., 2020; Table 1). CI causes embryonic death when males carrying an endosymbiont mate with females lacking it (or carrying a different strain). In *Wolbachia*, two‐gene (*cif*) operons cause CI, with male *cifB* expression (and occasionally *cifA*) killing embryos unless a complementary *cifA* copy is expressed in females (e.g., Beckmann et al., 2017; LePage et al., 2017; Sun et al., 2022). CI is regularly described as a trait whereby endosymbionts “manipulate” host reproduction—including by papers from our own groups—and the presence of the CI phenotype has led many to label *Wolbachia* (or particular variants of *Wolbachia*) as “manipulators” and even “parasites” (this terminology has been used for years in a variety of systems, for recent examples, see Beckmann et al., 2019; Kaur et al., 2021; Wybouw et al., 2022).

Cytoplasmic incompatibility serves as a particularly useful example of how imprecise language could lead to confusion and potentially incorrect conclusions. It is clear from theory that CI‐causing *Wolbachia* must increase host fitness to spread from low initial frequencies to become common (Hoffmann et al., 1990), with stochastic effects often expected to prevent *Wolbachia* spread (Jansen et al., 2008). CI is frequency dependent such that it has no effect on *Wolbachia* spread until *Wolbachia* surpass a tipping‐point equilibrium frequency in host populations (Turelli & Hoffmann, 1995) after which CI drives *Wolbachia* to usually stable high equilibrium frequencies balanced by rates of imperfect transmission (Hoffmann et al., 1990; Turelli & Hoffmann, 1995). This contrasts to *Wolbachia* that cause no or only weak CI, and occur at intermediate and often temporally (Cooper et al., 2017; Hague et al., 2020) and/or spatially variable frequencies (see 19). Weak selection on CI is supported by the common decay of *cif* operons that cause it (Beckmann et al., 2021; Martinez et al., 2021; Meany et al., 2019; Shropshire et al., 2023). For this reason, the relatively high incidence of CI in insect populations may have more to do with interclade selection (Turelli et al., 2022), which is supported by evidence of relatively recent acquisition of many *Wolbachia* endosymbionts in well‐studied systems (Cooper et al., 2019; Raychoudhury et al., 2009; Shropshire et al., 2023; Turelli et al., 2018). Selection on alternative *cif* functions may also indirectly contribute to the preservation of CI. This conjecture is supported by the preservation of particular domains (e.g., nucleases) that suggests Cif moonlighting (Kaur, Leigh et al., 2022; Terretaz et al., 2023). Notably, in other systems like *Cardinium*, CI expression occurs in the absence of *cif* homologs (Mann et al., 2017), highlighting that well‐understood endosymbiont phenotypes may have a distinct genetic basis and that genetic data alone are insufficient to infer putative phenotypes or to classify endosymbionts.

So does the CI trait represent a “manipulation” in the classic sense where the endosymbiont's trait effect helps it to spread despite a cost to its host? When the endosymbiont is at a very low frequency, CI makes no contributions to spread, with spread relying on other positive trait effects that are not “manipulations.” These dynamics underlie the weak purifying selection on CI and mutational disruption of *cifs* described above. Once the endosymbiont reaches an appreciable frequency, CI then contributes (along with these other positive trait effects) to spread; and only once the endosymbiont has spread to a high frequency can selection act to reduce CI strength. Thus, we only observe the CI trait in nature and its later contribution to spread because of other positive endosymbiont trait effects. While our argument is based on population dynamics, and one could argue CI is a “manipulation” at the trait level under some conditions, to refer to CI as “manipulation” in the classic sense does not seem particularly useful in trying to understand these complex dynamics. Clearly, referring to CI‐causing endosymbionts as “manipulators” is not useful since these endosymbionts have a net positive effect on host fitness.

What about non‐CI effects on reproduction like the induction of male killing (MK), parthenogenesis, and feminization? MK may represent a good example of “manipulation” in some circumstances. For instance, in *Adalia* ladybirds, male killing associated with *Wolbachia* endosymbionts results in female larvae gaining nutrition from the consumption of male eggs, increasing the number of ladybirds carrying *Wolbachia* at the expense of overall host egg production, and accounting for the female‐biased sex ratio (Hurst et al., 1993). However, the presence of any MK also seems insufficient to conclude that the associated endosymbiont represents a reproductive “manipulator” where endosymbiont transmission is favored at the expense of host fitness. Because of sib competition or a reduction in inbreeding, a host may also benefit from an MK phenotype, while the endosymbiont may also provide other host advantages such as *Spiroplasma* inducing protection against parasitoids (Xie et al., 2014). This suggests that while some endosymbionts producing MK may be accurately defined as “manipulators,” this classification is context‐dependent and needs additional data beyond showing the presence of an MK phenotype.

Parthenogenesis‐inducing endosymbionts in haplodiploids can increase host reproductive output (Segoli et al., 2013; Stouthamer et al., 1990), much in the same way as parthenogenetic lineages that do not involve endosymbionts. It is difficult to see these direct trait effects of endosymbionts on host fitness as examples of “manipulation” to overcome costs of being parasites, and perhaps, these bacteria themselves are better described as mutualists under specific conditions. Under feminization, the rate of transmission of an endosymbiont is increased due to feminized hosts being able to vertically transmit the endosymbiont; however, the host might also benefit from these endosymbionts in terms of increased reproductive output, although this is further complicated by incomplete endosymbiont transmission, incomplete feminization, and the fact that males can prefer genetic females over feminized females (Ferdy et al., 2016).

Endosymbionts also do not necessarily exhibit phenotypic effects on their hosts that might be expected to evolve from them being “manipulators.” For example, *Wolbachia* are expected to “modify” host behavior to favor their vertical transmission and increase their spread, such as through making *Wolbachia*‐carrying females more attractive to mates than females without *Wolbachia*. However, there is little evidence for these types of behavioral changes (Jiggins et al., 2002; Sullivan & Jaenike, 2006); in *D*. *melanogaster*, experiments designed to specifically test for such changes in mating preference failed to observe it (Arbuthnott et al., 2016), with studies reporting random mating or detecting apparent mating effects that are not easily repeated (de Crespigny & Wedell, 2007; Hoffmann et al., 1990). The exception to this pattern appears to be MK whose expression can be modified by the evolution of nuclear suppression of MK (Arai et al., 2020; Hornett et al., 2022; Richardson et al., 2023). This would imply that hosts lacking endosymbionts producing MK have a fitness advantage, consistent with the notion of the MK trait representing a true “manipulation.” The fitness advantage associated with suppressor genes may be particularly large if males are at a low frequency in a population due to a high frequency of MK‐inducing endosymbionts.

Although the evolution of suppressor genes of MK may be taken as evidence supporting the notion that the MK trait represents a true manipulation (Reynolds et al., 2019; Richardson et al., 2023), this phenomenon also highlights the substantial effects that the host can have on endosymbiont‐associated phenotypes (which continue to evolve through time). Classifying endosymbionts as manipulators underplays these host effects and the changing dynamics of hosts and their endosymbionts. As noted above, CI strength typically varies widely as a function of *Wolbachia*, host, and environmental variation, and these factors can result in evolution toward weaker CI. Stability of endosymbionts in populations is as much about transmission through hosts as any reproductive “manipulation” (Dyer & Jaenike, 2004; Turelli & Hoffmann, 1995). Hosts may even evolve a preference for mating with males without *Wolbachia* to reduce transmission, as suggested in spider mites (Vala et al., 2004).

## 4 THE MULTIPLE FACTORS INFLUENCING ENDOSYMBIONT DYNAMICS

**FIGURE 3:**
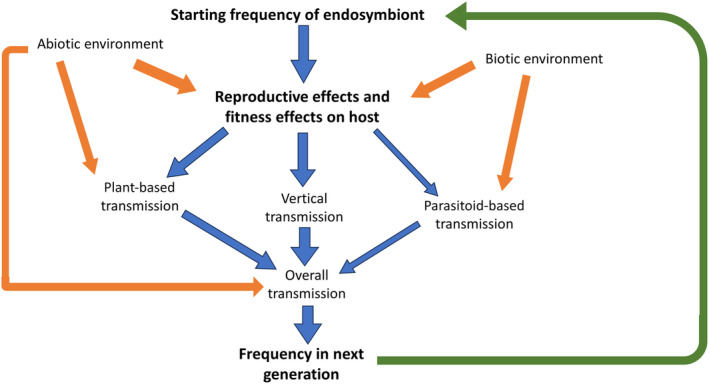
Processes influencing the dynamics of endosymbionts in natural environments. The reproductive and fitness effects of endosymbionts are embedded within abiotic and biotic components of the environment which also affect transmission processes.

Definitions of endosymbiont–host associations are often made with incomplete information—for example, using estimates of only one or a few traits from one or a few environments. This is not only insufficient to accurately place endosymbioses within the parasitism–mutualism continuum (Figure 1) but is also insufficient to capture the diverse processes that influence the dynamics of endosymbionts in natural populations (Figure 3). There is rich set of population, species, and environmental interactions that dictate the outcomes of endosymbiont–host associations. We highlight the different transmission processes that not only involve the host reproductive organs (through reproductive effects and vertical transmission) but also the biotic and abiotic components highlighted above. Moreover, both transmission and fitness effects can involve third parties. These include the plants attacked by herbivorous arthropods used for transmission by some bacterial endosymbionts capable of avoiding the plant immune system. Endosymbionts can also influence the interaction between plants and host fitness, with some bacteria capable of affecting arthropod effector systems that influence the efficiency of host feeding (Sharma et al., 2021). The abiotic environment impacts fitness, transmission, and indirectly reproductive effects, such as through the induction of self‐incompatibility if bacterial densities decline. This extends well beyond reproductive host effects which are important but need to be balanced against other phenotypic effects of endosymbionts.

The simplest dynamics apply to endosymbionts with little or no impact on host reproduction but with efficient vertical transmission. These are typified by *w*Au *Wolbachia* of *D*. *simulans* that transmits with near complete fidelity but does not generate reproductive effects on hosts (Hoffmann et al., 1996). Other transmitted endosymbionts may also have little phenotypic impact on hosts like *Rickettsia* in lacewings (Sontowski et al., 2020) and the *w*Mau *Wolbachia* variant of *D*. *mauritiana* (Meany et al., 2019). Thus, these may currently constitute nearly neutral endosymbiont systems, both in terms of fitness and reproduction as included in Figure 2. To allocate an endosymbiont to a neutral or non‐neutral category depends on the collection of phenotypic data across many host and environmental contexts, rather than relying on data from a single environment or on molecular genetic analyses.

By emphasizing effect sizes of endosymbionts in different contexts, the impacts of the host genetic background, environmental conditions, and presence of other organisms become the focus of investigation of endosymbionts. Unfortunately, there can be a disconnect between laboratory data that predict dynamics and the actual dynamics of associations in nature, making it challenging to identify the most important factors influencing endosymbiont prevalence in host populations. For instance, Smith et al (2021) expected an association between the frequency of *Hamiltonella* in parasitoid activity in field pea aphid populations based on reduced laboratory‐based parasitism due to the presence of this endosymbiont, but this was not borne out by frequency changes under field conditions. Field studies on another aphid also demonstrated no detectable effects of *Hamiltonella* on parasitism rates (Lenhart & White, 2017). Zepeda‐Paulo and Lavandero (2021) provide another example of a disconnect between rapid changes in the frequency of *Regiella* endosymbionts in *Sitobion avenae* aphids and parasitoid activity in cereal crops.

The performance of *Aedes* mosquitoes artificially associated with *Wolbachia* for biocontrol applications highlight challenges involved in understanding endosymbiont dynamics. Unassisted invasion of *Wolbachia* often does not occur in field sites despite initial expectations of empiricists based on the presence of CI in the laboratory (Garcia et al., 2019; Pinto et al., 2021). The spread and stability of *Wolbachia* in these systems explicitly depends on the environment, which can influence CI strength and the fidelity of maternal transmission (Ross et al., 2019). Factors affecting field frequencies of endosymbionts require intense studies of transmission, CI levels, and field fitness which have only been completed in a few cases such as for *Wolbachia* in *Drosophila* (Hague et al., 2020; Turelli & Hoffmann, 1995). The experience with *Wolbachia* transferred into mosquitoes is that dynamics can be dominated by local conditions where invasion is achieved at some sites, but endosymbiont frequencies are reduced at other nearby sites (Nazni et al., 2019).

## 5 CONCLUDING REMARKS

Endosymbiont dynamics are clearly challenging to capture with simple or single terms (Table 2). As we have noted for the case of “manipulation,” endosymbionts expressing CI must increase host fitness to initially spread, yet the field largely considers CI‐causing variants as parasites. Like many of our colleagues, we have used the term “manipulate” (e.g., Kriesner et al., 2016; Cooper et al., 2019; Richardson et al., 2023) and others like “hijack” (Shropshire et al., 2022) to describe CI (Table 2), but it seems clear to us now that this inaccurately describes the relevant endosymbiont–host dynamics. As we have noted, these trait effects may depend on host and environmental contexts, and well‐understood trait effects in one system may have independent molecular genetic bases in others (Shropshire et al., 2020). Thus, precisely describing the relevant genetic variation and molecular mechanisms underlying focal traits in their specific contexts will almost always be better than forcing them into single terms. A recent example is the term “hologenome” that forces the genomes of the host and associated microbes into a single genomic unit. As clearly noted by others, this term and broader framework may add little more than confusion to the field (Douglas & Werren, 2016; Moran & Sloan, 2015).

**TABLE 2:** Examples of unclear terms used in the endosymbiont literature and some alternatives.

Term(s)	Assumed placement on continuum	Possible risk of misinterpretation	Problems and consequences	Alternative
Addiction	Intermediate	Moderate	Implies unproven evolutionary process and fitness associations, anthropomorphism	Hosts “obtain resources” from endosymbiont for a specific function which may have evolved (evolutionary dependency)
Habit	″	″	″	″
Harboring	Mutualism or parasitism	Moderate	Host benefits from endosymbiont that it harbors or there is a cost, anthropomorphism	As above and with a clear indication of host fitness impacts
Infecting and infection	Depends on definition of infection and whether it refers to harmful effects or only the process of infection	Minor	Can imply negative fitness effects on host regardless of other impacts and applying the term to vertical transmission can also be problematical	Endosymbionts “are present” or “occupy niches” in hosts, or hosts “carry” endosymbionts
Intimate association	Mutualism	Moderate	Implies positive interactions, anthropomorphism	“Closely associated”
Partners or Partnership	″	High	″	″
Manipulation (usually refers to reproduction)	Parasitism	High	Classifies endosymbionts toward the parasitic end of the continuum, leads to other endosymbiont effects being ignored, anthropomorphism	Describe all phenotypic effects of endosymbiont, along with their magnitude and context
Hijacking	″	″	″	″
Protection	Mutualism	Moderate	Implies directed endosymbiont activity and mutualism which may not have been demonstrated	Endosymbionts “decrease” parasitoid mummification rates
Selfishness	Parasitism	Moderate	Implies intentional endosymbiont action toward the parasitic end of the continuum, which may be unproven	Compare impact of trait effects on fitness of host and transmission of endosymbiont
Symbiont	Mutualism	Limited	Dictionary definitions of symbionts imply mutualism, but the term now has a broader definition in biology	Alternative not likely needed but these are typically “host‐associated microbes”

Field‐specific shorthand using very standard terms can also create barriers to understanding. For example, we regularly describe CI as the reduction in observed egg hatch when uninfected females are crossed with “infected” males (Cooper et al., 2017). “Infection” is used to describe associations that span the parasitism–mutualism continuum, up to and including endosymbionts described as beneficial (Sachs, Essenberg & Turcotte, 2011). Yet as our colleagues have pointed out to us, “infection” has a very clear medical use and meaning that ties it to disease and harmful host effects (Casadevall & Pirofski, 2001).

Finally, we would argue that describing the local and more general dynamics of endosymbionts can also be improved by reducing unnecessary anthropomorphic terminology (Table 2). For example, cases where host functions are influenced by, but are not improved or expanded by, microbes have been described as a host “addiction” (Moran, 2002) or “habit” (Douglas, 2021). As described by Hammer (2023), “I need coffee to perform basic functions, but I do not perform them any better now than before the addiction began. I need coffee just to get back to normal. The same process can occur with host–microbe symbioses: a dependence evolves without an improvement in functionality.” While it is tempting to anthropomorphize discussions of endosymbiont–host associations in this way, using “addiction” seems unhelpful for several reasons. Definitions of “addiction” vary widely, complicating its application to endosymbiont–host associations. In the case of endosymbionts, their associations with hosts need not be chronic (i.e., endosymbionts come and go on short and long timescales), restricting the set of systems where “addiction” may even apply since persistent association is a precondition for “addiction” (Hammer, 2023). Hosts do not compulsively seek out endosymbionts to fill a need, which “addiction” generally implies (c.f. Sullivan, 2017). Instead, there may be an evolved dependency of endosymbionts and their hosts over a long‐time scale (Douglas, 2011), which can be accurately described in the context of the host and the environment without summarizing the interaction as an “addiction.”

Some of these terms remain very useful when used correctly; for example, “infection” can represent a precise descriptor of novel associations when microbes invade and multiply in new hosts, which represents a process (Peterson, 1996). Other terms like “manipulate” are less likely to be useful in the absence of additional context and without additional data being collected. We acknowledge the broader discussion on the language of science, and arguments for “night language” that that leaves room for anthropomorphisms that can help communicate important messages to the public, students, and other professionals (Yanai & Lercher, 2020). However, as noted by Harrison (2012), *the words we choose to describe concepts*, *models*, *patterns*, *and processes often reflect a particular outlook or point of view*…*the language we use can intentionally or inadvertently direct and constrain our thinking* (*and the thinking of others*). We agree and argue that imprecise (e.g., “manipulation”) and unnecessarily anthropomorphic (e.g., “addiction”) terms detract from understanding the complexity of endosymbiont–host interactions and their dependence on the ecological and evolutionary context in which they occur. Describing observed endosymbiont effects in the context of the host and its environment is a simple solution.

## AUTHOR CONTRIBUTIONS

**Ary A. Hoffmann:** Conceptualization (equal); methodology (equal); visualization (equal); writing – original draft (equal); writing – review and editing (equal). **Brandon S. Cooper:** Conceptualization (equal); methodology (equal); visualization (equal); writing – original draft (equal); writing – review and editing (equal).

## CONFLICT OF INTEREST STATEMENT

The authors declare no competing interests.

## Data Availability

There are no data included in this manuscript.
